# In vivo evaluation of a new hybrid graft using retrograde visceral perfusion for thoracoabdominal aortic repair in an animal model

**DOI:** 10.1016/j.xjtc.2022.07.022

**Published:** 2022-08-08

**Authors:** Sabine Wipper, Harleen K. Sandhu, Tilo Kölbel, Anthony L. Estrera, Constantin Trepte, Christoph Behem, Charles C. Miller, E. Sebastian Debus

**Affiliations:** a Department of Vascular Surgery, Medical University Innsbruck, Austria; b Department of Cardiothoracic and Vascular Surgery, McGovern Medical School at UTHealth, Houston, Tex; c Department of Vascular Medicine, University Heart Center Hamburg-Eppendorf, Germany; d Department of Anaesthesiology, University Hospital Hamburg Eppendorf, Germany

**Keywords:** SPIDER technique, hybrid aortic repair, Thoracoflo, thoracoabdominal aortic repair, TAAA, CO, cardiac output, CT, celiac trunk, ECC, extracorporeal circulation, FM, fluorescent microspheres, LRA, left renal artery, MBP, mean arterial blood pressure, OAR, open aortic repair, OP, operative, RRA, right renal artery, SMA, superior mesenteric artery, SVR, systemic vascular resistance, TAAA, thoracoabdominal aortic aneurysm, TTFM, transit-time flow measurement

## Abstract

**Objectives:**

The SPIDER technique for hybrid thoracoabdominal aortic aneurysm repair can avoid thoracotomy and extracorporeal circulation. To improve technical feasibility and safety, the new Thoracoflo graft, consisting of a proximal stent graft connected to a 7-branched abdominal prosthesis, was evaluated in a pig model for technical feasibility testing, before implantation in humans.

**Methods:**

Retroperitoneal exposure of the infradiaphragmatic aorta, including visceral and renal arteries, was performed in 7 pigs (75-85 kg). One iliac branch was temporarily attached to the distal aorta to maintain retrograde visceral and antegrade iliac perfusion after deployment of the thoracic stent graft segment (SPIDER technique). The proximal stent-grafted segment was deployed in the thoracic aorta via direct aortic puncture over the wire without fluoroscopy. The graft was deaired before flow via the iliac side branch to the visceral and iliac arteries was established. Visceral, renal, and lumbar arteries were subsequently sutured to the corresponding side branches of the graft. Technical feasibility, operating and clamping time, blood flow, and tissue perfusion in the related organs were evaluated before implantation and after 3 and 6 hours using transit-time flow measurement and fluorescent microspheres. Final angiography or postprocedural computed tomography angiography were performed.

**Results:**

Over-the-wire graft deployment was successful in 6 animals without hemodynamic alteration (*P* = n.s.). In 1 pig, the proximal stent graft section migrated as the guidewire was not removed, as recommended, before release of the proximal fixation wire. Angiography and computed tomography scan confirmed successful graft implantation and transit-time flow measurement confirmed good visceral and iliac blood flow. Fluorescent microspheres confirmed good spinal cord perfusion.

**Conclusions:**

Over-the-wire implantation of the Thoracoflo graft using the SPIDER technique is feasible in a pig model. No fluoroscopy was required. For safe implantation, it is mandatory to follow the single steps of implantation.

**Figure:**
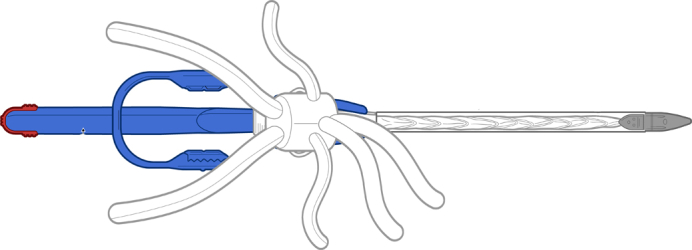
Thoracoflo graft prototype with proximal ring-shaped stent graft.

**Central Message**

The Thoracoflo graft for TAAA hybrid repair shows promising results in animal testing. However, its potential to reduce perioperative mortality and morbidity must be confirmed in a clinical setting.

**Perspective**

The Thoracoflo graft is designed for hybrid TAAA repair, avoiding thoracotomy and ECC by using the previously established SPIDER technique. The graft design was modified and reevaluated to improve graft deployment, fixation and stability. Feasibility testing, including quantitative assessment of organ perfusion, was performed in an in vivo experimental setting before first implantation in human.

Based on the Thoraflex hybrid graft (Vascutek Ltd), the Thoracoflo graft was developed as a new hybrid device for thoracoabdominal aortic aneurysm (TAAA) repair. The aim was to offer preventive strategies concerning perioperative mortality and morbidity by combined endovascular thoracic and open abdominal aortic approach for TAAA repair in a single-stage operation. The SPIDER technique for graft implantation allows temporary retrograde distal perfusion of visceral and renal arteries via the iliac access side branch following the deployment of the thoracic stent graft of the device. Hereby, thoracotomy and extracorporeal circulation (ECC) can be avoided while enabling reimplantation of visceral, renal, and lumbar arteries.^1^^,^^2^ Based on first clinical experience using an off-the-shelf Thoraflex hybrid-graft for TAAA repair, the SPIDER graft was specifically designed for retrograde delivery into the descending thoracic aorta.^3^ After early feasibility testing in an experimental setting in a pig model, the graft design was further modified and reevaluated to improve graft deployment, fixation and stability, and deairing, to enable reattachment of lumbar arteries and to establish over-the-wire implantation.^4^

This report describes evaluation of the modified graft design and implantation technique of the new Thoracoflo graft for TAAA repair in an experimental setting in pigs, including quantitative assessment of visceral and spinal cord perfusion.

## Methods

Seven domestic pigs with a mean body weight of 80 ± 5 kg were operated on at the animal laboratories, University Heart Center Hamburg. The study was performed in accordance with the “Position of the American Heart Association on Research Animal Use” (*Circulation*, April 1985) and approved by the government animal care committee (AZ101/15) and the institutional review board for the care of animal subjects.

### Device Description

The Thoracoflo graft consists of a proximal stent graft with ring stents and a distal gelatin-sealed polyester multibranched graft. First prototypes (SPIDER graft) were previously described and tested in an experimental setting, confirming further need of modification to improve intraoperative handling, graft stability, to enable reattachment of lumbar arteries, and over-the-wire implantation.^1^^,^^4^

**Figure 1:**
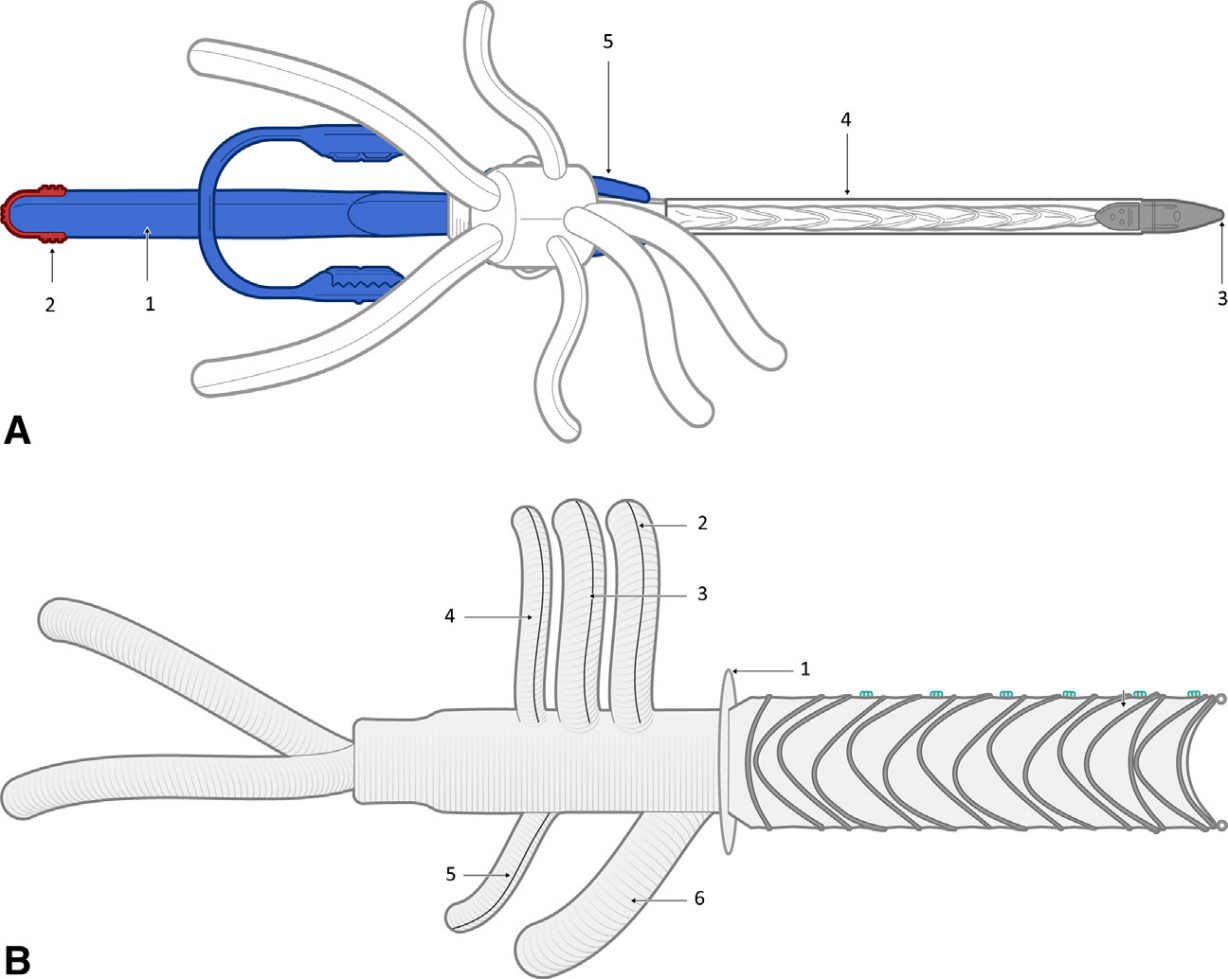
A, Thoracoflo graft prototype with proximal ring–shaped stent graft connected to 7-branched gelatin-sealed Dacron graft for reattachment of visceral and renal arteries, iliac arteries, and reimplantation of lumbar arteries, in between collar for graft fixation. The handle (1) with release clip (2) for the fixation wire of the proximal stent graft component on the introducer tip (3), equipped with a side hole for introduction of the guidewire, is outsided through the 10-mm access branch. The stent graft is loaded in a splitable 30-F peel-away sheath (4) and fixed with a splitter (5). B, Deployed Thoracoflo graft prototype shown from the dorsal side: thoracic stent graft (24 mm diameter, 150 mm length): collar (1) at connection to Y-shaped arm for biliary reattachment and 4 prosthetic arms for reimplantation of celiac trunk (2); superior mesenteric artery (3); and both renal arteries (4, 5). The access branch (6) was used as loop graft for reimplantation of lumbar arteries.

The Thoracoflo graft prototype (Figure 1, *A* and *B*) in the experimental setting for graft evaluation was adjusted to the aortic diameters of an 80-kg pig observed from postmortem computed tomography scan. The gelatin-sealed polyester prosthesis consists of a long, Y-shaped arm (8 mm each side) for biliary reattachment and 4 prosthetic arms (8 mm for visceral, 6 mm for renal arteries) attached for reimplantation of celiac trunk (CT), superior mesenteric artery (SMA), and both renal arteries (left renal artery [LRA], right renal artery [RRA]). A 10-mm access branch with a length of 20 mm carries the handle with the delivery system and is positioned on the dorsal side of the main body for reattachment of lumbar arteries after graft deployment. Between the stent-grafted thoracic component and the multibranched, gelatin-sealed polyester prosthesis, a Siena collar is included, enabling suture fixation to the native aortic vessel to prevent stent graft migration after deployment.

The stent graft is 150 mm in length and 24 mm in diameter. Longitudinal stiffness and stability of the stent graft are achieved by special orientation and formation of the ring stents. The stent graft section is loaded into a 30-F peel-away sheath. The tip at the top of the delivery system is equipped with a side hole, enabling over-the-wire implantation of the graft via direct puncture of the aorta. The handle with the delivery system is externalized via the 10-mm access branch on the dorsal side of the main body of the multibranched graft and fixed with a splitter. The elongated design of the splitter prevents the amount of blood loss during extraction of the delivery system through the access branch. The release wire for fixation of the proximal stent graft component on the introducer tip is inserted through the delivery system and fixed on a release clip at the end of the handle.

### Device Implantation

**Figure 2:**
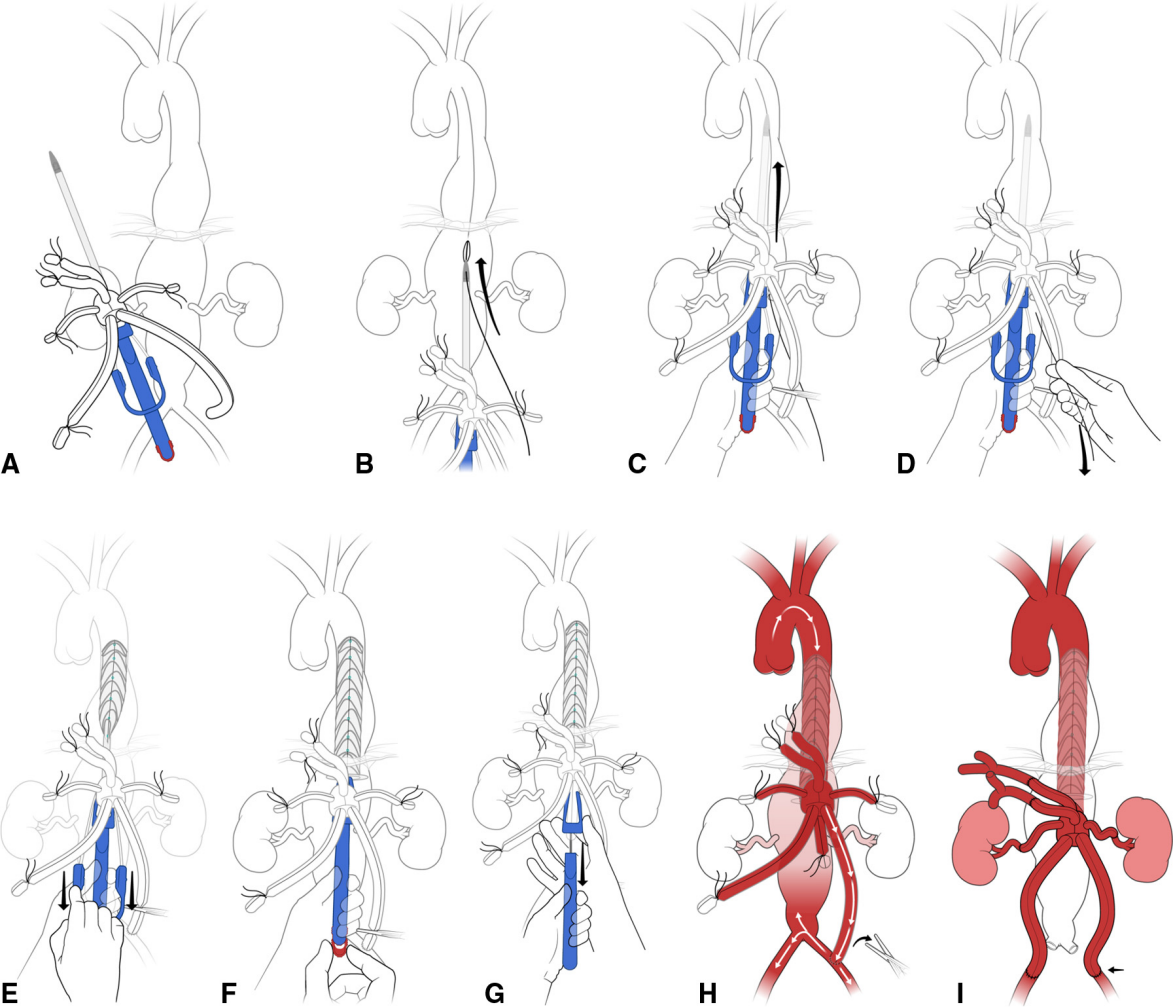
Technical steps of Thoracoflo graft implantation. A, Ligation of contralateral iliac branch and visceral side and temporary connection of the iliac side branch to distal aorta or iliac artery in end-to-side fashion. Clamping of the iliac branch. B, Puncturing of the aorta close to the coeliac trunk ostium and guidewire introduction. C, Over-the-wire introduction of the stent-grafted section of the Thoracoflo graft until the collar is just outside the puncture hole. Guidewire is outsided through a side hole of the tip. D, Retraction of the guide-wire. E, Retraction of the peel-way sheath. F, Release of wire for proximal fixation wire of the stent graft. G, Opening of splitter and removement of the handle through the access branch. H, Establishment of retrograde visceral perfusion by release of clamp on iliac branch. I, Subsequent reattachment of visceral and renal arteries and reattachment of lumbar arteries.

The single steps of Thoracoflo graft implantation are illustrated in both Figure 2 and Video 1. The abdominal aorta, including CT, SMA, LRA, and both iliac arteries, was exposed via a retroperitoneal approach. Before implantation of the Thoracoflo graft, all side branches—besides the one for the CT—were distally ligated by 2-0 VICRYL suture (Ethicon Inc, a subsidiary of Johnson and Johnson). The left iliac branch was temporarily anastomosed end-to-side to the distal aorta or to the left common iliac artery and clamped until the stent graft was deployed and the graft was de-aired.

The stent-grafted section of the Thoracoflo graft was introduced over the guidewire after direct puncture of the aortic wall. Aortic puncture was performed at the level of the CT ostium and the guidewire was established into the descending thoracic aorta. The needle was retracted and the puncture hole was stepwise dilated using a 10-F and a 14-F dilator sheath, while cardiac output (CO) was reduced by in-flow reduction by pulling on a loop surrounding the inferior vena. The guidewire was externalized through the side hole in the nose cone of the introducer-tip. Then, the stent graft component of the hybrid graft was introduced until the collar was just outside the vessel wall. The peel-away sheath was retracted and the guide wire extracted before release of the proximal fixation wire and retraction of the handle.

The proximal part of the extended splitter was opened, allowing retraction of the delivery system with minimized blood loss, and the handle was carefully retracted until the nose cone extended into the side access branch. The splitter was completely removed, the handle was extracted, and the access branch was clamped. In-flow reduction was released, and the graft was deaired and flushed with heparinized saline solution via the access branch. Then, retrograde distal perfusion via the previously attached iliac side branch was established, hereby, avoiding ECC and thoracotomy. Fluoroscopy is not mandatory for thoracic graft deployment, as transesophageal echocardiography can be used during stent-graft introduction to ensure correct positioning of the graft. Thereafter, stepwise visceral and renal arteries were reattached in end-to-end fashion to the corresponding branches of the Thoracoflo graft starting with the CT, followed by SMA. The aorta is then crossclamped below the renal arteries and the Siena collar is sutured to the aortic wall using continuous 3-0 or 4-0 PROLENE suture (Ethicon Inc, a subsidiary of Johnson and Johnson) to prevent back-bleeding of intercostal arteries and distal graft migration. Thereafter, RRA and LRA are anastomosed.

Finally, lumbar arteries were reimplanted as patch into the previous 10-mm access branch, which can be used as loop graft by reattachment of the distal part of the branch to the left iliac side-branch. Reattachment of the lumbar arteries can be performed either as a loop graft or in end-to end fashion, depending on the individual surgeon's preference. In case no lumbar arteries are reattached, the introducer branch can be ligated.

The initial end-to-side anastomosis of the iliac branch was replaced by end-to-end anastomosis to both iliac arteries.

### Anesthesia and Instrumentation

After intramuscular premedication with azaperone (4 mg/kg), midazolam (0.3 mg/kg), ketamine hydrochloride (5 mg/kg), and atropine sulfate (0.15 mg/kg), intravenous anesthesia was induced by pentobarbital (8 mg/kg) and maintained by continuous infusion of fentanyl (0.01 mg/kg/h), midazolam (0.1 mg/kg/h), and ketamine hydrochloride (0.06 mg/kg/h). Pigs were endotracheally intubated and pressure-control ventilated at 15 cm H_2_O with a positive end-expiratory pressure of 7 cmH_2_O at 16 breaths per minute using 30% oxygen. Activated clotting time of at least 300 seconds was achieved by heparin (400 IU/kg). A 6-F sheath was inserted in the right carotid artery for monitoring of mean arterial blood pressure (MBP) and arterial blood-gas analyses. A Swan–Ganz catheter (Pulsion) was inserted via the right jugular vein for monitoring of central venous pressure, pulmonary arterial pressure, and left atrial pressure. The Swan–Ganz catheter was zeroed to ambient air pressure to calibrate before each measurement. A 5-F PiCCO catheter (Pulse Contour Cardiac Output; Pulsion Medical Systems) was inserted in the left common femoral artery to monitor CO, systemic vascular resistance, and distal MBP. The PiCCO catheter was calibrated at the beginning of each experiment using thermodilution with 20 mL of NaCl at 8°C (mean value of 3 consecutive injections). For quantitative assessment of organ perfusion using fluorescent microspheres (FMs), a 5-F catheter was introduced into the left atrium to enable FM injection, and a 5-F pigtail catheter is inserted into the descending thoracic aorta via a 7-F sheath in the common femoral artery for retraction of reference blood.

All hemodynamic and respiratory parameters were continuously monitored and recorded according to a standardized protocol at each intervention. Isotonic and colloidal solutions were continuously infused at a basic flow rate of 400 mL/h to achieve a stable global end-diastolic volume.

After retroperitoneal exposure of the abdominal aorta, visceral and LRA as well as both iliac arteries were dissected free from surrounding tissue to place a 6-mm flow-measurement probe (CardioMed; Medistim AS), to monitor the blood flow during measurements by transit-time flow-measurement (TTFM) technique.

### Experimental Protocol and Hemodynamic Measurements

As in the previous experimental studies, graft implantation was performed by the same 2 senior vascular surgeons with high experience in open aortic surgery. Hemodynamic parameters were continuously monitored and recorded during each intervention. Arterial and venous blood-gas analyses and TTFM were acquired before implantation (baseline), after stent-graft deployment and establishment of retrograde visceral perfusion using SPIDER technique for proof of concept of retrograde visceral perfusion, after implantation of the prosthesis with anastomosis of all side branches (operative [OP]), and hourly during an observation time of 6 hours. Final angiogram was performed after 6 hours of observation using the 5-F Pigtail catheter in the descending thoracic aorta.

Quantitative assessment of liver, kidney, bowel, and spinal cord perfusion by FMs was performed during baseline, after implantation (OP), and at 6 hours. At the end of the experiments, the pigs were humanely killed by intravenous injection of 20 mL of T61 under deep anesthesia. The Thoracoflo graft was explanted for photo documentation. Two randomly selected representative animals underwent postmortem computed tomography angiography using a 12-F sheath in the distal aorta and the inferior vena cava for postoperative quality control. Finally, one lobe of the liver, both kidneys, 30 cm of bowel, and spinal cord were harvested and preserved for at least 10 days in 4% formalin for later FM assessment.

### Fluorescent Microspheres

As previously described, FM (15 μm in diameter; Molecular Probes), were injected into the left atrium during withdrawal of a reference blood sample via the descending thoracic aorta, for quantitative assessment of organ perfusion.^1^^,^^4^ To ensure proper mixing before injection, each vial of microspheres was placed in an ultrasonic water bath for 5 to 10 minutes and vortexed twice for 3 minutes. During each measurement, approximately 3 × 10^6^ FMs were suspended in physiological saline solution to a volume of 10 mL and injected constantly over 60 seconds at each intervention. Reference blood samples were withdrawn from the descending thoracic aorta through the previously established 50-F pigtail catheter with a constant-rate withdrawal pump at 3.14 mL/min over 3 minutes (model 640A; Harvard Apparatus). At the end of the experiments, the pigs were humanely killed, and one representative lobe of liver, both kidneys, 30 cm of bowel, and spinal cord were harvested and fixed in 4% formaldehyde for at least 10 days. Visceral organs were dissected into 6 tissue pieces weighing 4 g each. Spinal cord was dissected in total length into pieces of equal size weighing between 2.5 and 3 g each. Tissue samples and arterial blood reference were processed for determination of blood blow by spectrofluorometry, according to the standard method described by Glenny and colleagues.^5^

### Statistical Analysis

Descriptive statistics for continuous variables were calculated using univariate moments of the distributions (median and interquartile range) at each of the 3 time points (baseline, operative, and 6-hours' postoperative), with the exception of operative and ischemic times, which are total times reported only once per pig. Tests for variation by time point for hemodynamic and flow measurements were computed using mixed models with a fixed effect for time and a random effect for subject to account for the correlation of repeated measures within pig. Because multiple measures per pig per time point were made for microsphere tissue perfusion assessments, these data were analyzed using hierarchical mixed models that included multilevel random effects for sample nested in pig with repeats by time point. The null hypothesis was rejected at a nominal alpha of *P* < .05. All computations were performed using SAS software, version 9.4 (SAS Institute).

## Results

Thoracoflo graft implantation was successful in 6 of 7 pigs, including 6-hour follow-up without hemodynamic alteration.

### Technical Feasibility

Retroperitoneal exposure of the abdominal aorta, including visceral, renal, and iliac arteries, was performed without significant blood loss or hemodynamic events in all 7 animals. Over-the-wire introduction of the Thoracoflo graft after direct aortic puncture next to the CT ostium was fast, easy, and comfortable to perform after dilatation of the puncture side under in-flow reduction. Retraction of the peel-away sheath, extraction of the release wire for proximal fixation of the stent graft, and removal of the splitter were technically easy to perform.

In one pig, the guidewire was not removed, as recommended before release of the proximal fixation wire and retraction of the handle, resulting in distal migration and collapse of the proximal stent-grafted section of the Thoracoflo graft. This pig was excluded from further investigation to evaluate the Thoracoflo graft.

No stent-graft migration occurred in the remaining 6 pigs when the guidewire was released, as recommended before release of the proximal fixation wire. Retraction of the handle with the elongated splitter of the device required no additional surgical maneuvers or manual help. Deairing of the graft was easy to perform via the introducer branch, but modification of the splitter—including a closure mechanism for the introducer branch to avoid the need for manual branch occlusion during retraction of the handle and to avoid accidental blood loss—was favored by both surgeons implanting the graft.

Reattachment of visceral, iliac, and lumbar arteries showed favorable anatomical orientation of the visceral side branches. Both kidney arteries in the pig model were very small compared with the visceral arteries—and very sensitive toward vasospasm during the anastomosis to the 6-mm branches of the graft. Hemodynamic stability was maintained in all 6 animals throughout Thoracoflo graft implantation and during 6 hours of observation. Intraoperative angiography and postprocedure computed tomography angiography confirmed open-side branches and successful graft implantation in all 6 animals but mismatch in the diameter for the attached kidney arteries.

### Implantation and Ischemic Times

Data are shown as median (interquartile range). Graft implantation lasted 75 minutes (65-83 minutes). Implantation time of the stent-grafted section was 4.5 minutes (4.5-4.5 minutes). Ischemic time of the visceral organs was minimized to the time needed for the anastomosis of the respective side branch and lasted 15 minutes (13-16 minutes) for CT and 8 minutes (8-9 minutes) for SMA. Ischemic time of left kidney was added to right kidney ischemic time due to simultaneous crossclamping of both renal arteries during opening the juxtarenal aortic segment. RRA was first anastomosed from inside. Ischemic time for the RRA was 13 minutes (12-14 minutes) and for left kidney was 26 minutes (21-35 minutes).

### Hemodynamic Parameters and Organ Perfusion

Hemodynamic stability was maintained in all 6 animals throughout the implantation and the 6-hour observation period. While diastolic blood pressure and consecutively MBP decreased during graft implantation and 6-hour follow-up period compared with baseline (*P* < .05), systolic blood pressure, CO, central venous pressure, and global end-diastolic volume did not significantly change throughout implantation and 6 hours observation compared with preoperative baseline values (*P* = n.s.). No catecholamines were used to increase diastolic blood pressure throughout the protocol, as systolic blood pressure was stable. MBP was successfully decreased by 50% from baseline during aortic puncture, dilatation of the puncture side, and stent graft implantation, until retrograde distal perfusion via the previously attached iliac side branch was established. Relevant hemodynamic parameters during baseline, graft implantation, and 6-hour observation are illustrated in Table 1. No significant blood loss occurred during the implantation, as confirmed by stable hemoglobin levels (*P* = n.s.).

**Table 1:** Hemodynamics

Variable	Baseline	Operative	6-h post	Model SE	*P* value OP vs BL	*P* value OP vs 6 h	*P* value 6 h vs BL
SBP	105.7	108.5	104.8	5.9	<.641	<.548	<.891
DBP	64.0	50.7	48.0	4.3	<.012	<.550	<.004
MAP	77.9	69.9	66.9	4.0	<.072	<.466	<.020
CVP	9.5	7.0	8.8	1.3	<.080	<.183	<.615
CO	7.2	7.6	6.2	0.7	<.525	<.055	<.161
GEDV	1106.3	1110.8	1026.3	39.3	<.912	<.058	<.070

Values are mixed-model least-squares means. *Model SE*, Common standard error for contrasts; *Op vs BL*, *P* for operative versus baseline; *OP vs 6 h*, *P* for operative versus 6-hours post; *6 h vs BL*, 6-hour post versus baseline; *SBP*, systolic blood pressure; *DBP*, diastolic blood pressure; *MAP*, mean arterial pressure; *CVP*, central venous pressure; *CO*, cardiac output; *GEDV*, global end-diastolic volume.

### Transit-Time Flow Measurement (TTFM)

TTFM confirmed sufficient retrograde visceral perfusion by passive shunting using SPIDER technique: TTFM flow for CT was 545 (450-700) mL/min, for SMA 620 (535-780) mL/min, and for LRA was 225 (210-260) mL/min. Due to retroperitoneal aortic access, there was no TTFM value for RRA. After implantation and during 6-hour follow-up, TTFM flow of visceral, renal, and iliac arteries confirmed sufficient blood flow to the visceral arteries. TTFM flow for CT was stable during implantation (*P* < .038) but significantly increased during 6-hour observation compared with baseline (*P* < .050). SMA flow increased significantly during implantation (*P* < .037) and was stable compared with baseline after 6-hour observation (*P* < .112). The LRA showed significantly lower values compared with baseline after implantation and throughout the observation, reflecting the vasospasm and the mismatch in diameter of the sensitive porcine kidney arteries to the corresponding branches of the graft (*P* < .05). The TTFM values during baseline, implantation (OP), and postoperative observation (6 hours) are illustrated in Table 2.

**Table 2:** Transit time flow measures

Variable	Baseline	Operative	6-h post	Model SE	*P* value OP vs BL	*P* value OP vs 6 h	*P* value 6h vs BL
Celiac trunk	570.0	678.3	800.0	102.9	<.318	<.265	<.050
SMA	647.5	855.8	798.3	83.3	<.037	<.521	<.112
Left renal	217.5	121.5	99.3	24.0	<.003	<.378	<.001
Left iliac	418.3	493.3	258.3	94.4	<.446	<.033	<.122
Right iliac	390.0	360.0	260.4	44.4	<.489	<.052	<.018

Values are mixed-model least-squares means. *Model SE*, Common standard error for contrasts; *OP vs BL*, *P* for operative versus baseline; *OP vs 6 h*, *P* for operative versus 6-hours post; *6 h vs BL*, 6-hours post versus baseline; *SMA*, superior mesenteric artery.

### Quantitative Assessment of Tissue Perfusion (FM)

FM application for quantitative assessment of visceral and spinal cord perfusion was successful in all 6 animals during baseline, after OP, and after 6 hours of observation. Six specimens of equal size (3.9 ± 0.2 g each) of the formalin-preserved segments of liver and both kidneys were dissected and processed for fluorospectrometry. The spinal cord was dissected in toto into 9 to 15 samples, weighing 3.7 ± 0.3 g each, depending on the lengths and thickness of the spinal cord. Liver perfusion decreased significantly during implantation (*P* < .02) but increased during 6-hour observation to baseline values (*P* < .264). Bowel perfusion showed no significant differences throughout implantation and observation time (*P* = n.s.). FM confirmed reduced kidney perfusion compared with baseline. Spinal cord perfusion remained stable compared with baseline after implantation (*P* < .693) and during 6-hour observation (*P* < .101). Rate of change of organ perfusion showed no treatment-by-time interactions for change from baseline to 6-hour postimplantation. Table 3 summarizes TTFM values of liver, bowel, kidney, and spinal cord perfusion.

**Table 3:** Tissue perfusion—microspheres

Variable	Baseline	Operative	6-h post	Model SE	*P* value OP vs BL	*P* value OP vs 6 h	*P* value 6 h vs BL
Liver	0	–44.3	143.4	47.6	<.002	<.037	<.264
Bowel	0	36.5	14.5	10.6	<.287	<.040	<.312
Left kidney	0	–61.9	–75.9	3.8	<.001	<.677	<.001
Right kidney	0	–29.7	–43.4	3.5	<.001	<.001	<.001
Spinal cord	0	–1.5	19.4	10.1	<.693	<.043	<.101

Values are mixed-model least-squares means, given as percent change from baseline, since perfusion per gram of tissue is not straightforward to interpret clinically, although *P* values are computed from untransformed data to meet model assumptions. *Model SE*, Common standard error for contrasts; *OP vs BL*, *P* for operative versus baseline; *OP vs 6 h*, *P* for operative versus 6-hours post; *6 h vs BL*, 6-hours post versus baseline.

## Discussion

The SPIDER technique using the new Thoracoflo graft for hybrid TAAA repair aims to avoid ECC and thoracotomy and minimize ischemic time of visceral organs while enabling reimplantation of lumbar arteries to reduce risk of spinal cord ischemia: after lumbotomy, the Thoracoflo graft is inserted retrogradely for descending thoracic aortic repair, with subsequent anastomoses between the connected multi-branched Dacron graft and the renovisceral arteries and the iliac outflow, respectively.

The graft was successfully implanted in 6 of 7 pigs. For technical success, it is essential to follow the single steps of implantation, as recommended. One pig violation of the implantation protocol resulted in distal graft migration and collapse of the stent graft, as the guidewire was not removed before pulling back the handle. FM results documented stable spinal cord perfusion throughout the implantation and during the 6-hour observation period but also demonstrated impaired kidney perfusion compared with the relatively increased bowel and liver perfusion in the experimental setting. The implanting surgeons predicted potential kidney malperfusion due to the anatomical mismatch in size and location in the nondiseased porcine model. Nevertheless, angiogram and computed tomography angiography confirmed open renal arteries in all pigs. In humans, the renal arteries are larger and less sensitive toward vasospasm, and the anatomy for reattachment is more favorable in an aneurysmatic patient.

In the pig model, substitution of vasoactive medication, such as norepinephrine, was avoided, as SBP and CO were stable, to avoid the impact on spinal cord perfusion. Increasing the MBP might have improved renal perfusion compared with the increased perfusion of bowel and liver. However, the anatomy of the porcine spinal blood supply is markedly different from that of humans. Also the segmental thoracic and lumbar arteries are relatively small and often originate as a single branch from the aorta.^6^

The Thoracoflo graft was designed to improve perioperative mortality and morbidity during TAAA open repair. Fenestrated and branched endovascular repair for TAAA have undergone tremendous innovations within the last decade and are meanwhile routinely used in dedicated centers. Nevertheless, open aortic repair (OAR) remains the standard of care in many centers, especially in patients with genetic aortic syndromes. Despite this, OAR has undergone major improvements, such as multimodal access to organ protection, consisting of mild hypothermia, cerebrospinal fluid drainage, left heart bypass, sequential clamping of the aorta, and selective reimplantation of the intercostal as well as lumbar arteries. This complex procedure remains challenging and carries high morbidity and mortality, which globally has led to centralization of these highly demanding procedures.^7^, ^8^, ^9^, ^10^, ^11^, ^12^, ^13^, ^14^

While the aneurysm extent and rupture presentation are not modifiable risk factors, hypotension, blood loss, intraoperative clamp time, and patent lumbar and intercostal artery sacrifice are factors that can be potentially influenced. Avoiding ECC for selective visceral perfusion can reduce the perioperative risk of bleeding, the need for blood products, the inflammatory response, and the risk of ECC related hypotension. Preventing the risk of ECC, as well as ischemia reperfusion-related hypotension, might also reduce the risk of perioperative spinal cord ischemia.^15^, ^16^, ^17^ The Thoracoflo graft enables TAAA repair without needing ECC by using SPIDER technique for retrograde visceral perfusion via an iliac side branch. In our study, the proof of concept of the SPIDER technique for TAAA hybrid repair using the Thoracoflo graft was confirmed by TTFM measurement after implantation of the thoracic stent graft section and establishing blood flow to the visceral organs via the previously attached iliac side branch.

Intraoperative clamping time and ischemic times can be minimized using the SPIDER technique. Ischemic time is reduced to the time needed for each side-branch anastomosis added to the time needed for stent graft deployment. This was also confirmed previously by significantly shorter ischemic times compared with conventional OAR.^4^ The present study showed further reduction of the ischemic time compared with the previous graft design by using direct aortic puncture without the need for clamping and dissection the CT ostium during stent graft deployment.

The Thoracoflo graft enables reattachment of lumbar arteries, which might reduce the risk of spinal cord ischemia compared with endovascular techniques that lack this option. Quantitative assessment of spinal cord perfusion by FM showed stable spinal cord perfusion throughout the entire 6-hour observation period. This was also confirmed in a previous study of our group.^4^ In our experimental setting, only lumbar arteries were reattached and perfusion measurements were performed. For further evaluation of neurologic outcomes, intraoperative neurologic monitoring, such as somatosensory-evoked potentials and motor-evoked potentials, and a longer postoperative follow-up period with neurologic testing might be useful. In case of spinal cord ischemia, it might be an option to perform a thoracotomy for reattachment of the distal intercostal arteries. However, it might be challenging to reattach crucial intercostal arteries in the distal part of the descending aorta. In addition, technical difficulties might occur during the intraoperative management of back-bleeding from intercostal arteries arising from the descending thoracic wall.^18^ This has not been evaluated thus far in the experimental setting in healthy, nonaneurysmatic aortas.

By hybrid TAAA repair using the Thoracoflo graft thoracotomy and ECC might be avoided. This could significantly reduce pulmonary complications in the clinical setting, which are a major cause of morbidity and mortality.^19^ Thus, patients with TAAA may benefit from this novel approach, which minimizes the invasive trauma associated with thoracoabdominal exploration. Finally, no fluoroscopy or contrast agents are required during stent-graft deployment. However, in patients with dissection, the introduction of the guidewire should be performed under controlled conditions, using transesophageal echocardiography to ensure graft deployment in the true lumen. In the clinical setting, patients pretreated with thoracic endovascular aortic repair will be selected for the first cases to have a safe landing zone before patients with type IV aneurysm are selected. Therefore, patients with connective tissue disease pretreated with aortic arch repair using the frozen elephant trunk technique or conventional repair will enable the attachment of a thoracic endovascular aortic repair for patients of interest.

The Thoracoflo graft for TAAA hybrid repair shows promising results in experimental testing. However, the potential to reduce perioperative mortality and morbidity during open TAAA by avoiding the ECC and thoracotomy while enabling reattachment of lumbar arteries must be confirmed in the clinical setting.

### What the Paper Adds

The Thoracoflo graft is designed for hybrid TAAA repair, avoiding thoracotomy and ECC by using the previously established SPIDER technique. The graft design was modified and re-evaluated to improve graft deployment, fixation and stability, and deairing as well as to enable reattachment of lumbar arteries and establish over-the-wire implantation. Feasibility testing, including quantitative assessment of organ perfusion, was performed in an in vivo experimental setting before first implantation in human.

### Conflict of Interest Statement

Drs Estrera and Sandhu are consultants for W. L. Gore & Associates, Inc. Dr Estrera is also a consultant for CryoLife, Edwards Lifesciences, and Terumo Aortic. Drs Kölbel and Debus report intellectual property rights and travel grants with Terumo Aortic. All other authors reported no conflicts of interest.

The *Journal* policy requires editors and reviewers to disclose conflicts of interest and to decline handling or reviewing manuscripts for which they may have a conflict of interest. The editors and reviewers of this article have no conflicts of interest.

## Supplementary Data

Video 1 This video summarizes the design of the Thoracoflo graft, including the single steps of graft preparation and implantation. The graft can be introduced either after direct aortic puncture over the wire into the aorta or via the ostium of the coeliac trunk or superior mesenteric artery. For safety reasons, the size of the aortic access must be large enough to extract the handle of the graft after stent graft deployment. Therefore, the puncture side should be cut large enough to accommodate. Video available at: https://www.jtcvsorg/article/S2666-2507(22)00429-1/fulltext.
