# Supplementary figures and images for: In vivo evaluation of a new hybrid graft using retrograde visceral perfusion for thoracoabdominal aortic repair in an animal model
Source: JTCVS Tech. 2022 Aug 8;15:1–8. doi: 10.1016/j.xjtc.2022.07.022 (PMC9579856; doi:10.1016/j.xjtc.2022.07.022)

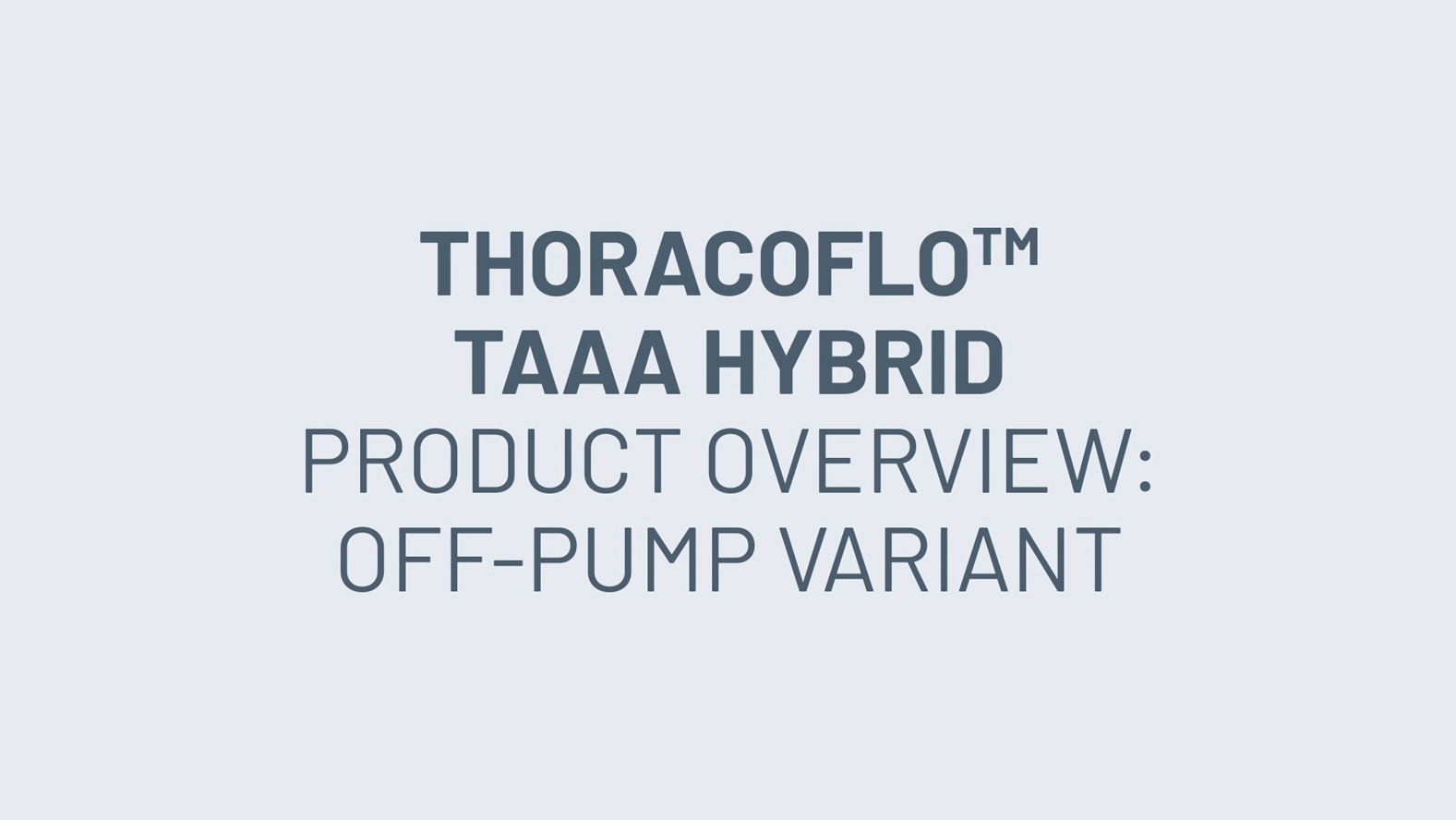

Supplement: Video 1 — This video summarizes the design of the Thoracoflo graft, including the single steps of graft preparation and implantation. The graft can be introduced either after direct aortic puncture over the wire into the aorta or via the ostium of the coeliac trunk or superior mesenteric artery. For safety reasons, the size of the aortic access must be large enough to extract the handle of the graft after stent graft deployment. Therefore, the puncture side should be cut large enough to accommodate. Video available at: https://www.jtcvsorg/article/S2666-2507(22)00429-1/fulltext. [file fx2.jpg]
